# Seahorse-Tail-Inspired Soft Pneumatic Actuator: Development and Experimental Characterization

**DOI:** 10.3390/biomimetics9050264

**Published:** 2024-04-27

**Authors:** Michele Gabrio Antonelli, Pierluigi Beomonte Zobel, Muhammad Aziz Sarwar, Nicola Stampone

**Affiliations:** Department of Industrial and Information Engineering and Economy (DIIIE), University of L’Aquila, P.le Pontieri 1, Località Monteluco, 67100 L’Aquila, Italy; gabrio.antonelli@univaq.it (M.G.A.); pierluigi.zobel@univaq.it (P.B.Z.); muhammadaziz.sarwar@graduate.univaq.it (M.A.S.)

**Keywords:** bio-inspired soft pneumatic actuator, soft robotics, seahorse tail, finite element analysis, experimental characterization

## Abstract

The study of bio-inspired structures and their reproduction has always fascinated humans. The advent of soft robotics, thanks to soft materials, has enabled considerable progress in this field. Over the years, polyps, worms, cockroaches, jellyfish, and multiple anthropomorphic structures such as hands or limbs have been reproduced. These structures have often been used for gripping and handling delicate objects or those with complex unknown a priori shapes. Several studies have also been conducted on grippers inspired by the seahorse tail. In this paper, a novel biomimetic soft pneumatic actuator inspired by the tail of the seahorse *Hippocampus reidi* is presented. The actuator has been developed to make a leg to sustain a multi-legged robot. The prototyping of the actuator was possible by combining a 3D-printed reinforcement in thermoplastic polyurethane, mimicking the skeletal apparatus, within a silicone rubber structure, replicating the functions of the external epithelial tissue. The latter has an internal channel for pneumatic actuation that acts as the inner muscle. The study on the anatomy and kinematic behaviour of the seahorse tail suggested the mechanical design of the actuator. Through a test campaign, the actuator prototype was characterized by isotonic tests with an external null load, isometric tests, and activation/deactivation times. Specifically, the full actuator distension of 154.5 mm occurs at 1.8 bar, exerting a maximum force of 11.9 N, with an activation and deactivation time of 74.9 and 94.5 ms, respectively.

## 1. Introduction

Humans have always been attracted to nature and its creatures. Bioinspiration precisely concerns the study of biological structures to understand their functioning and characterize their materials, the interactions between the constituent elements, and their replication. Studies in this area have made it possible to explain how evolutionary processes over billions of years have led various species to adapt to the most hostile environments efficiently and optimally. Not surprisingly, many scientists and engineers have often taken inspiration from nature itself for technological innovations. The study of spiders’ arctic relationships has enabled a rotary rolling diaphragm actuator [1]; understanding self-healing epithelial tissues has driven the search for materials to make skins capable of restoring their mechanical behavior [2,3]. In other cases, single human fingers have been made [4,5] or entire anthropomorphic, three-finger tendon-driven hands [6] or five-finger pneumatically actuated hands [7]. A gripper for granular materials of various sizes was made from the study of the grasping mode of the elephant’s trunk [8]. Inspired by the elephant trunk, a certain number of artificial muscle fibers provide for selective deformation in continuous movements [9].

With the advent of soft robotics, the reproduction of bioinspired structures has increased enormously, pushing it to an unexpected level compared to the past [10]. The two main drivers of this field of research have been both the availability of soft materials [11], which are easily deformable and inherently safe, and 3D printing, especially fused deposition modeling (FDM) [12], to make molds for injection silicone rubbers and to print actuators [13] directly. In several works, studies of different reinforcing structures of marine species are reported with the help of FDM technology; in particular, the potentialities of FDM are shown to study their mechanical behavior even when not disposing of natural specimens or when interested in making and testing structures that did not develop in nature [14,15].

In recent years, it has been possible to reproduce bio-inspired structures such as octopus tentacles [16,17], jellyfish with pulse-jet locomotion [18], soft appendages to make underwater robots [19], the structure of a cockroach [20], the movement and control of snakes [21] or dogs [22], worms for potential applications in colonoscopy [23], their locomotion in different environments [24,25], or the combination of soft airtight skin and a scissor structural skeleton [26,27].

Implementations of these capabilities are due to the spread of soft actuators, coming to equip robots with a wide variety of abilities in a safe, simple, and versatile way [28]. Often, these actuations are based on soft pneumatic actuators (SPAs), i.e., actuations by compressed air, which use the presence [4,29] or absence [30] of an external reinforcement to guide the deformation of a hyper-elastic deformable component. Indeed, it is possible to take advantage of specifically designed geometries either directly or by making deformable components externally reinforced.

Because of the strong nonlinearities due to the nonlinear behavior of compressed air, the nonlinear constitutive law of the hyper-elastic material, and the large deformations of the actuator, the finite element method (FEM) is often used for the analysis, design, and simulation of the performance of SPAs [31,32] or for topological optimizations [33].

**Figure 1:**
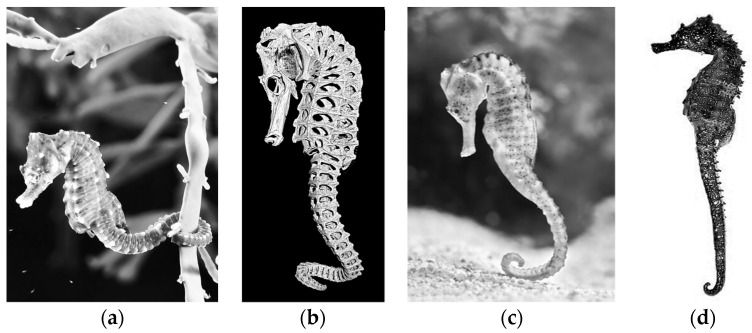
(**a**) *H. hippocampus* with its tail anchored to plant on seabed; (**b**) detail of plates and rings of *H. reidi* species; (**c**) detail of detaching from seabed; (**d**) seahorse with tail slightly bent backward.

The present work is in the context of bioinspiration, namely, the design, numerical simulation by the FEM, and prototyping of a SPA inspired by the tail of the seahorse *Hippocampus reidi*. The inspiration for the seahorse is due to the high number of degrees of freedom of its tail, which is an excellent context for making a soft prototype. Regardless of the species, seahorses, or *Hippocampus*, belong to the class of bony fishes and range in size from 1.5 to 35.5 cm. They are adept at camouflaging themselves in their habitats and swim very stunted, waving a dorsal fin and using their pectoral fins to guide. Because of this poor swimming dexterity, seahorses are often found on the seabed resting with their prehensile tails wrapped around seagrasses, mangroves, and coral reefs (Figure 1a) [34,35]. Their mass is up to 230 g, and although they are bony fish, they do not present scales but have smooth, thin, taut skin over a series of bony plates. Their bodies have prismatic bony plates, while their tails consist of square rings (Figure 1b). The skeleton of a seahorse’s tail is a multifunctional device that provides structural support, protection, and prehension. The tail can actively bend in the ventral direction (prehension), in the combined ventral-lateral direction (helical bending), and in the dorsal direction (overstretching) [36], enabling the seahorse to grasp and move objects or detach its body from the seabed (Figure 1c) [36,37]. A study has dealt with the tail’s anatomy, going so far as to explain why the outer geometry of the rings constituting it is square rather than cylindrical [37]. Several works have analyzed the mechanisms of tail deformation [37,38,39,40]. In other studies, a cobot gripper inspired by the seahorse tail structure was made for gripping objects [41,42]. Finally, soft robots for object grasping have been made, such as the soft spiral robots (SpiRobs) [43] and the square continuum robot (SCR) [44]. All the proposed devices are tendon-driven and electrically powered.

The proposed SPA is made of a 3D-printed reinforcement in thermoplastic polyurethane (TPU), mimicking the skeletal apparatus, with an R PRO20 silicone rubber structure that replicates the functions of the epithelial tissue. Inside the rubber structure is an air-feeding channel that acts as the inner muscle of the seahorse, equivalently as a pneumatic actuator. The choice of pneumatics is due to its ability to work economically and safely, avoiding motors moving the cables of a possible tendon structure and minimizing the weight of the actuator. A complete characterization was carried out for isotonic tests with an external null load, isometric tests, and activation/deactivation times. The purpose of this work is not the realization of an actuator for grasping objects but the development of a leg for sustaining a multi-legged robot inspired by the movement of the seahorse, which can support its weight with its tail, whose deformation will provide for generating forces for the locomotion. The conceived idea of the robot expects four supporting limbs, just like the one characterized in this work. By independently adjusting the feeding pressure, the deformation of each actuator can be managed to generate the force necessary for locomotion. The latter can be carried out by the alternative powering of two actuators at a time, e.g., the left front one and the right rear one, and then reverse and pressurize the right front one and the left rear one while the discharge of the previous ones occurs.

The following items represent the originality of this work:

- The design and fabrication of an actuator pneumatically powered and inspired by the *Hippocampus reidi*;
- The methodology to achieve an integration between skeleton and epithelial tissue, as for several biological structures in nature;
- A complete characterization of the designed SPA.

The paper is organized as follows: Section 2 describes the mechanical design of the proposed actuator, passing through details on the *Hippocampus reidi* species, analyzed in this study, the CAD modeling, and numerical simulations; in Section 3, the manufacturing process to realize the SPA is detailed; and the experimental characterization and discussion of the prototyped actuator are reported in Section 4.

## 2. Mechanical Design of the Bio-Inspired Actuator

### 2.1. The Studied Species: Hippocampus reidi

**Figure 2:**
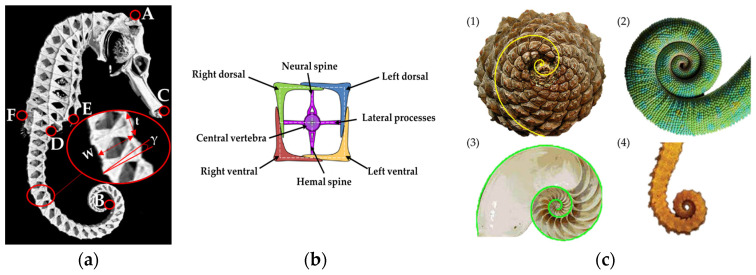
(**a**) Detail of seahorse dimensions: fish height (A–B, with stretching tail), head length (A–C), tail length (D–B, with stretched tail), maximum width (E–F), and constituent elements of tail segment with its characteristic dimensions (*W*, *t*, *γ*); (**b**) detail of the components of tail segment; (**c**) examples of logarithmic spiral in different natural structures: (1) pinecone, (2) chameleon tail, (3) shell, and (4) seahorse tail.

The seahorse *H. reidi* is one of the 35 known seahorse species in the world. The choice of this species is mainly due to the scientific community’s interest in it and, consequently, the anatomical resources available for its bio-inspired modeling [36,37]. This species has an average height of 14.4 cm, reaching specimens up to 19.2 cm, considering the head, the trunk, and the tail. The skeleton, made of vertebras, absorbs shock and protects the inner muscle that passes and runs from the top of the head to the end of the tail, within each vertebra. Along the trunk and tail, bony segments are approximately equidistant, while at the end of the tail, the segments appear close together. The seahorse can independently manage the folding of each segment from the others. The tail consists of a chain of squared segments that decrease in size (width, *W*, and thickness, *t*), while the skew (*γ*) increases as they move along the tail (Figure 2a).

Each segment turns out to consist of four L-shaped prismatic elements (left dorsal, right dorsal, left ventral, right ventral), held together by a vertebra. The central amphicoelous vertebra is made of a hemal spine, a neural spine, and two lateral processes [42] (Figure 2b). The dorsal and ventral spines are the only spine tails that are not involved in the formation of a joint. Each segment contains 13 joints: 8 act like gliding joints; the remaining 5 act like ball-and-socket joints [39].

The tail is often held ventrally curled and is flexible. When forced, it can slightly bend backward (Figure 1d) and, when coiled forward, follows a logarithmic spiral, as several existing structures in nature (Figure 2c).

### 2.2. Characteristic Dimensions of the Actuator

**Figure 3:**
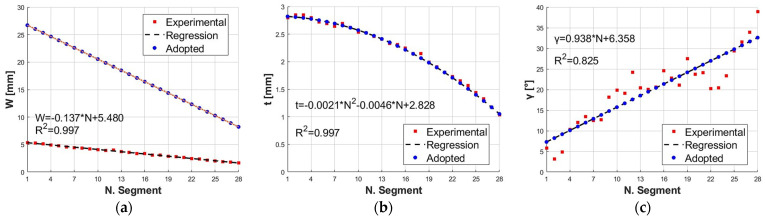
Dimensions of the *H. reidi* segments: (**a**) experimental and adopted *W*; (**b**) experimental and adopted *t*; (**c**) experimental and adopted *γ*.

Through studies on the size of the specimens of *H. reidi* [40], it was possible to express the values of *W*, *t*, and γ of each segment of the tail as a function of the number of segments, *N*. As shown in Figure 3a–c, *W* and *t* decrease as the number of sectors progresses, while *γ* increases. The identified trends, by a regression for *W*, *t*, and *γ* as a function of *N*, are reported in Equations (1)–(3):

(1) W=−0.137·N+5.480

(2) $$t=-0.0021\cdot N^{2}-0.0046\cdot N+2.828$$

(3) γ=0.938·N+6.358

As for the actuator to be developed, it is necessary to scale up *W* to realize the SPA by a molding process, print the reinforcement by the FDM, and assemble them easily and correctly. The values of *t* and *γ* follow Equations (2) and (3). Based on the identified regression trends and setting a scaling factor F equal to 5, the adopted values of *W* (Figure 3a) were obtained by the following equation:

(4) W=F·(−0.137·N+5.480)

**Figure 4:**
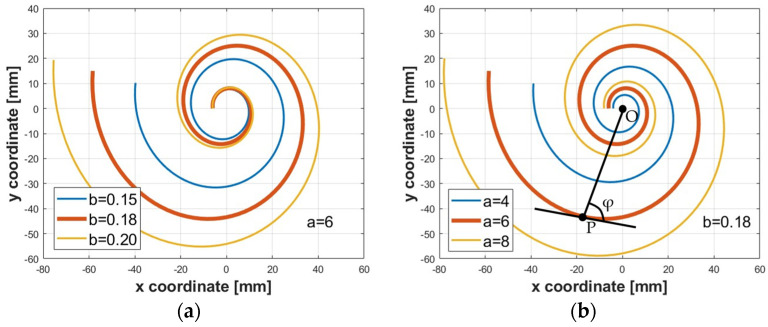
Details of logarithmic spirals: (**a**) *a* = 6 mm and *b* equal to 0.15 (blue line), 0.18 (orange line), 0.20 (yellow line); (**b**) *b* = 0.18 and *a* equal to 4 mm (blue line), 6 mm (orange line), 8 mm (yellow line). The thick orange line represents the spiral adopted in the present work.

Moreover, the starting and the final radii of the logarithmic spiral were estimated. The logarithmic spiral can be described by Equation (5):

(5) $$r=a\cdot e^{b\cdot \theta },$$

where *a* is a scaling factor, *b* is the cotangent of the constant polar tangential angle *φ*, defined as the angle between the tangent of a point P belonging to the spiral and the line connecting the point to the origin O, as shown in Figure 4, and θ is the angle of rotation as the curve spirals. In a cartesian coordinate system, for $\theta \in \left[0,\;4\pi \right]$, the coordinates of the points belonging to the spiral can be expressed by Equations (6) and (7):

(6) x(θ)=r·cos⁡(θ)

(7) $$y\left(\theta \right)=r\cdot sin(\theta )$$

The constants *a* and *b* were set equal to 6 mm and 0.18, respectively. These values were chosen to avoid collisions between the edges of the segments of the bio-inspired actuator. The midpoint of each segment coincides with a point of the spiral. Specifically, to show the influence of these parameters, different logarithmic spirals were constructed for a value of *a* fixed at 6 mm with various values of *b* (Figure 4a) and for a value of *b* fixed at 0.18 with various values of *a* (Figure 4b).

### 2.3. CAD Model of the SPA

**Figure 5:**
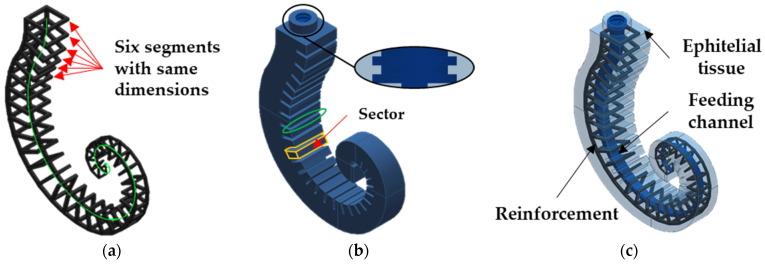
CAD model: (**a**) reinforcement with 33 segments connected along the dorsal side and a detail about the guiding logarithmic spiral profile (green line); (**b**) the covering inspired by epithelial tissue with the detail about labyrinth gaskets (black circle) and a virtual hinge (green ellipse); (**c**) the assembled SPA.

The modeled SPA consists of a reinforcement inspired by the seahorse tail skeleton and a covering inspired by its epithelial tissue. A feeding pneumatic channel, acting as the inner muscle of the seahorse, is realized within the covering. The reinforcement wraps and contains the radial deformation of the pressurized feeding channel, maximizing the actuator elongation. For modeling the three components, the logarithmic spiral identified in the previous Section was adopted as a guiding profile (with a limited *θ* in $\left[0,\;2.5\pi \right]$), shown as a green line in Figure 5a. In the same figure, the reinforcement with an overall amount of 33 segments is shown. The first six segments have the same dimensions (*W* = 26.75 mm, *t* = 2.8 mm) as the first segment identified in the previous Section, while the remaining twenty-seven follow the trends reported in Figure 3a–c. The segments were connected only on the dorsal side to allow for agile SPA elongation. Moreover, each segment consists of a single squared element, joining the 4-L-shaped prismatic elements and not considering the four processes that are present in the biological tail.

The reinforcement was overlaid with a 6 mm thickness of coating on all sides, as shown in Figure 5b, to be fully embedded inside the covering. The latter also shows cutouts (green-colored ellipse, Figure 5b) in the ventral part that act as virtual hinges and fall between two adjacent segments of the reinforcement. The covering between two consecutive cutouts is called a sector (volume highlighted by yellow-colored lines, Figure 5b). The feeding channel acts as a pneumatic actuator: it allows the spiral to be unrolled, extending the SPA in the case of positive pressures, or to be rolled up, flexing the SPA in the case of negative pressures. A connector equipped with a pneumatic fitting provides for the inlet/outlet of the air into the feeding channel. 

In Figure 5b, details about labyrinth gaskets to ensure the pneumatic and mechanical seal between the connector and the SPA are reported. Figure 5c shows the modeled assembly. The assembled 3D geometry of the actuator was modeled in Solid Edge 2024.

### 2.4. The Numerical Model

It was developed to check the functionality of the proposed actuator and, hence, proceed to the prototyping of it. Numerical models were implemented by the commercial Ansys Workbench 2023 R2 code, working with four cores in parallel. A PC with a 2.2 GHz 8th-generation intel i7 8750H processor, an NVIDIA GTX 1050 Ti GPU, and 32 GB of RAM was adopted. The 3D geometry of the actuator was exported into the FEM code. However, a boolean subtraction operation was performed in the FEM code to remove all the volume occupied by the reinforcement from the epithelial tissue. Bonded contacts were applied between the outer surfaces of the reinforcement and the inner ones of the epithelial tissue. Simulations were conducted on only half of the geometry, including symmetry constraints, to minimize the computational time and cost.

The modeled components of the actuator are the R PRO20 coating and TPU reinforcement structure. R PRO20 is a Shore A 21-hardness silicone rubber with an elongation at a break of 250%. The mechanical behavior of the silicone rubber was modeled with the 2-parameter Mooney–Rivlin model, C_01_ = 0.0628 MPa, C_10_ = 0.0694 MPa, D_1_ = 0.61 MPa^−1^, and Poisson’s ratio of 0.46, experimentally achieved. TPU was modeled as an isotropic elastic material with a Young’s modulus of 50 MPa and a Poisson’s ratio of 0.4. The reason for choosing isotropic behavior lies in the small deformations of the reinforcement when the actuator operates.

**Figure 6:**
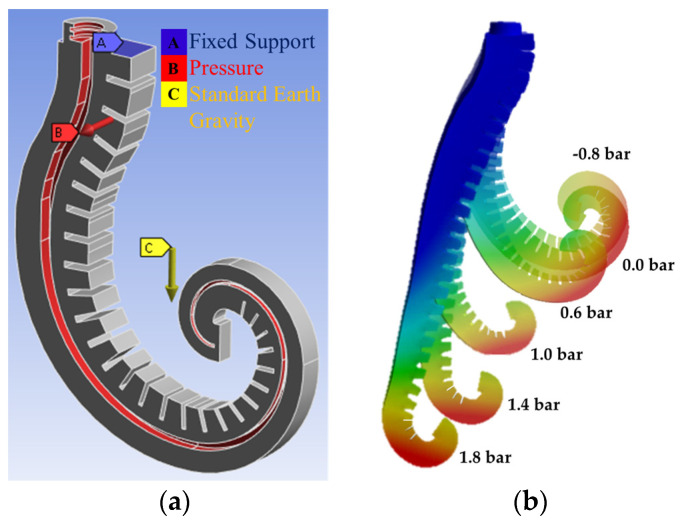
The FEM model of the actuator: (**a**) constraints and boundary conditions; (**b**) deformation results at different pressure levels.

The following common settings were adopted in all numerical models: automatic meshing with elements less than 2.8 mm in size (106,431 nodes and 67,754 elements); tetrahedral elements for modeling all components; a fixed constraint at the top end of the covering (blue-colored Fixed Support A, Figure 6a); a pressure ramp in the range (−0.85–1.8) bar acting on the surfaces of the feeding channel (red-colored areas Pressure B, Figure 6a) in the simulated time interval 0–1 s; gravitational effects (yellow-colored Standard Earth gravity C, Figure 6a); bonded contact constraints based on the penalty stiffness method between the reinforcement and the cover; nonlinear analysis by the Newton–Raphson method; large deformation algorithm; and auto time stepping, controlled by the program.

Figure 6b shows the deformations resulting from the action of the feed pressure at −0.8 bar, 0.0 bar (only gravitational effects), 0.6 bar, 1.0 bar, and 1.8 bar. Results show that it is possible to adjust the extension and flexion of the SPA by pressure regulation. The results in Figure 6b are only qualitative, but a quantitative comparison between numerical and experimental results is in Figure 12b,c.

## 3. The Prototype

For construction needs, the reinforcement must be realized and directly attached to the core for the feeding channel. Hence, the reinforcement was designed to be rigidly connected by removable structural attachments to the core. They were prototyped in TPU by a QIDI i-fast 3D printer. The chosen layer thickness of 0.1 mm is compatible with the 0.4 mm nozzles used. A low printing speed, set equal to 40 mm/s, and an inner and outer wall speed of 20 mm/s, for an extruder temperature of 220 °C and the printing plate at 65 °C, avoid warping and limit stringing. The filling percentage was set at 100% according to a concentric configuration.

**Figure 7:**
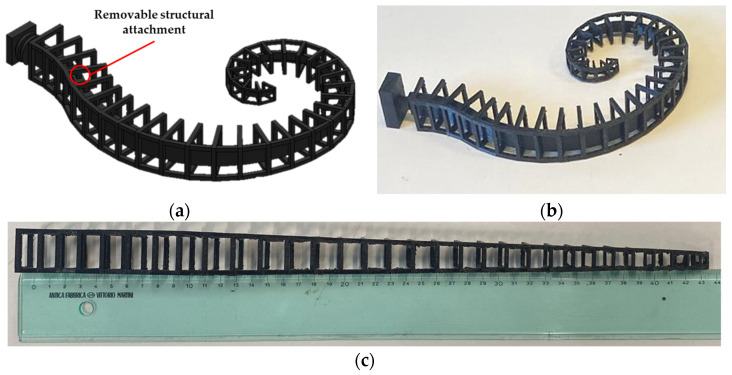
The reinforcement in TPU: (**a**) designed reinforcement with the core; (**b**) prototyped reinforcement with the core; (**c**) fully unrolled reinforcement (perspective effects are visible in the figure).

The designed reinforcement with the core and the refined one, after cleaning and printing support removal, are shown in Figure 7a,b, respectively. To demonstrate the easy deformability of the reinforcement without the core, as it occurs in the functioning of the actuator, and to measure its overall plane development length, a 3D print without the core was carried out. As shown in Figure 7c, the reinforcement can be fully unrolled, and its length measures 435 mm.

The silicone rubber covering was made by injection inside a 3D-printed PLA mold. After accurately mixing parts A (base) and B (catalyst) of the R PRO20 silicone rubber in a ratio of 1:1, the mixture was cooled to delay solidification and put under a vacuum pump for the removal of air bubbles; then, it was put into the mold.

**Figure 8:**
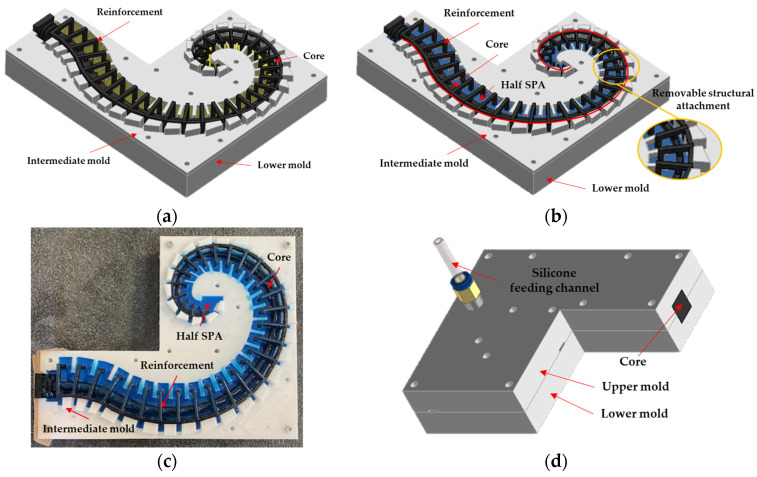
SPA manufacturing process: (**a**) stage I: mold assembly with the cavity to be filled (in yellow); (**b**) stage I: half SPA realized (blue), the link between intermediate mold and reinforcement (along red line) and the detail about reinforcement–core connection (yellow circle); (**c**) experimental top view of the end of stage I; (**d**) stage II: the intermediate mold is replaced with the upper mold, and silicone rubber is injected by a double-acting cylinder.

Concerning Figure 8, the mold is made of three components: the lower mold, the intermediate mold, and the upper mold. The molding process was divided into two stages:

- stage I: for the realization of the lowest half of the actuator. The reinforcement and core are suspended in the lower mold by attachments mounted in the intermediate mold, as shown in Figure 8a. In the same figure, the yellow surface represents the area to be filled by silicone rubber. The latter, the blue-colored volume in Figure 8b, is poured from above into the lower mold up to the interface surface between the two molds. In Figure 8b, there are also details about the connection between the reinforcement and intermediate mold (red line) and the reinforcement–core connections that ensure the proper distance between them (yellow circle). Figure 8c shows the experimental end of the first stage;
- stage II: for the realization of the remaining half of the actuator. After 3 h at 20 °C, the intermediate mold must be removed. The attachments between the reinforcement and core must be cut. This operation allows the remaining part of the reinforcement and the core to be surrounded by silicone rubber, and the core can be removed in the final stage to obtain the feeding channel. It was no longer necessary to keep the reinforcement and core raised, as the solid silicone rubber on the bottom acts as a support. Hence, the upper mold replaces the intermediate one. Figure 8d shows the mounting of the overall set of molds with the pneumatic fitting used to feed the silicone by an injection process with a double-acting cylinder.

**Figure 9:**
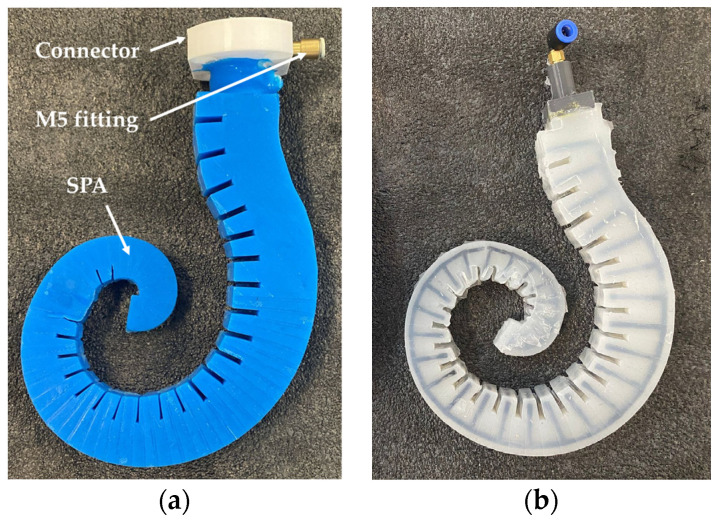
The prototype of the SPA: (**a**) the prototype made of R PRO20 adopted for the present work; (**b**) a similar prototype for showing the reinforcement within the covering.

After 3 h at 20 °C, the complete solidification of the silicone rubber occurs, and the prototype is removed from the molds. The operation is completed with the extraction of the core, the removal of the silicone burrs, and the assembly of the connector. The prototype of the actuator is shown in Figure 9a. In Figure 9b, a similar actuator was realized by another type of silicone rubber, which is not useful for the aim of the present work, to show how the reinforcement (black lines) is included within the covering (translucent white structure).

The connector for the air inlet/outlet was printed by a 1.75 mm wire diameter of PLA with a layer height of 0.2 mm, a 100% infill, a printing speed of 100 mm/s, an extruder temperature of 210 °C, and the printing plate temperature constant at 60 °C. After inserting the connector, a plastic clamp, later covered by the same silicone rubber, must be externally applied in correspondence with the gasket seals previously mentioned.

The overall mass of the prototype, measured by A CS-2000 balance (resolution ± 0.1 g), is 473 g. In detail, the M5 pneumatic fitting has a mass of 7 g, the air inlet/outlet port of 18 g, and the SPA of 448 g.

## 4. Experimental Characterization

The prototype was experimentally characterized to achieve the pressure–deformation relationship with a null external load acting on it, the exerted force–pressure relationship at different deformation initial rates, and the activation/deactivation times.

**Figure 10:**
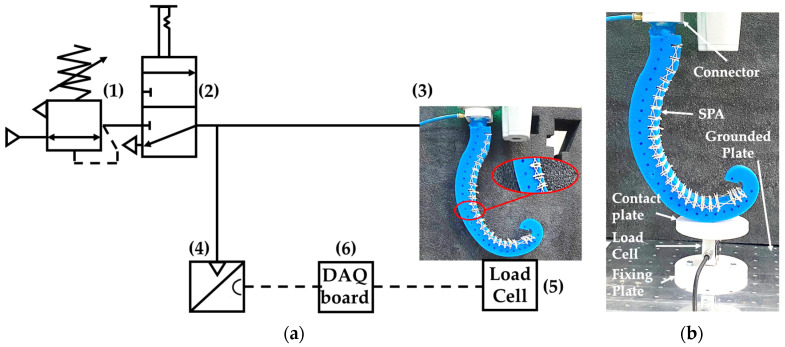
The test bench: (**a**) a schematic with the components and details about PLA plates (red circle) to limit the radial deformation and markers (blue points in the red circles); (**b**) a detail of the actuator placed by the cobot on the load cell.

In all the tests, the prototype was attached by its connector to the wrist of the OMRON TM5-700 cobot, set in collaborative function mode [45], and adopted to adjust the placement of the actuator according to the test requirements. In addition, to limit the radial deformation of the SPA, as also carried out in [46], a couple of interconnected PLA plates (thickness 1.0 mm) were placed on the lower and upper surfaces of each sector, as shown in Figure 10.

The same test bench was adopted for each of the tests. It is made of the components reported in Figure 10a: (1) an SMC IR1220-N01-A precision pressure regulator (maximum pressure of 4.0 bar) to adjust the pressure within the actuator, (2) a pneumatic 3/2 bistable valve with manual lever operation (PNEUMAX CLD 228.32.9/2) as a pneumatic on/off valve, (3) the actuator prototype, (4) a Honeywell ABPMANN004BGAA5 analog pressure transducer (full range 4.0 bar, output signal 0.5–4.5 Vdc, accuracy ±0.25% of full scale, response time 1 ms) to measure the feeding pressure value, (5) an analog AEP transducer TCA10 tension/compression load cell (full scale ±10 kg; output signal ±10 Vdc; nominal sensitivity 2 mV/V; sensitivity tolerance ≤ ±0.1%) to measure the exerted force by the actuator, and (6) a National Instruments USB-6001 data acquisition board, to acquire the signals of the pressure transducer and load cell. The load cell was mounted by a plastic fixing plate on a grounded aluminum plate; a plastic contact plate was mounted on the load cell to transmit the force exerted by the actuator to the load cell, as shown in Figure 10b. All of the components were adopted during the experimental activity, except the load cell, which was adopted only when force measurements were required.

### 4.1. Isotonic Tests with an External Null Load

**Figure 11:**
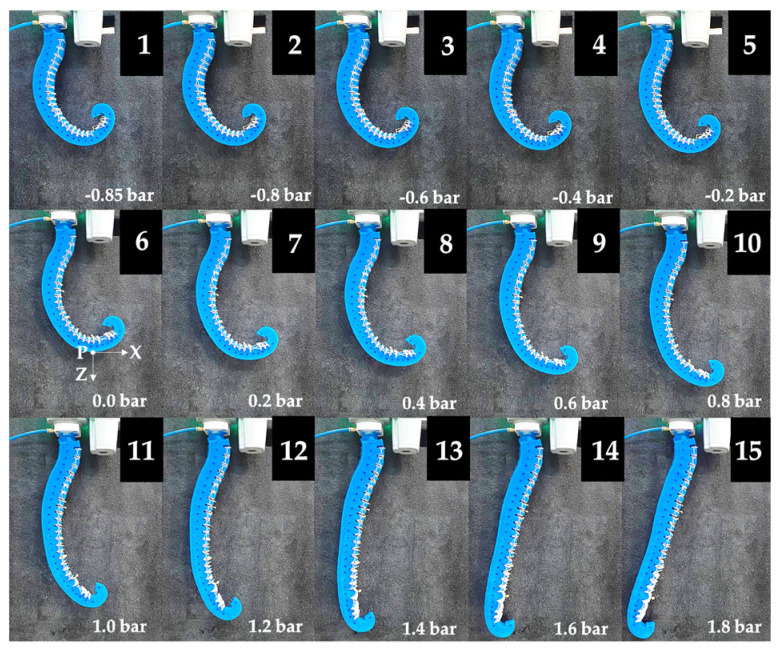
Deformations of the actuator in isotonic tests with a null external load as a function of the pressure at: (1) −0.85 bar; (2) −0.8 bar; (3) −0.6 bar; (4) −0.4 bar; (5) −0.2 bar; (6) 0.0 bar; (7) 0.2 bar; (8) 0.4 bar; (9) 0.6 bar; (10) 0.8 bar; (11) 1.0 bar; (12) 1.2 bar; (13) 1.4 bar; (14) 1.6 bar; (15) 1.8 bar.

**Figure 12:**
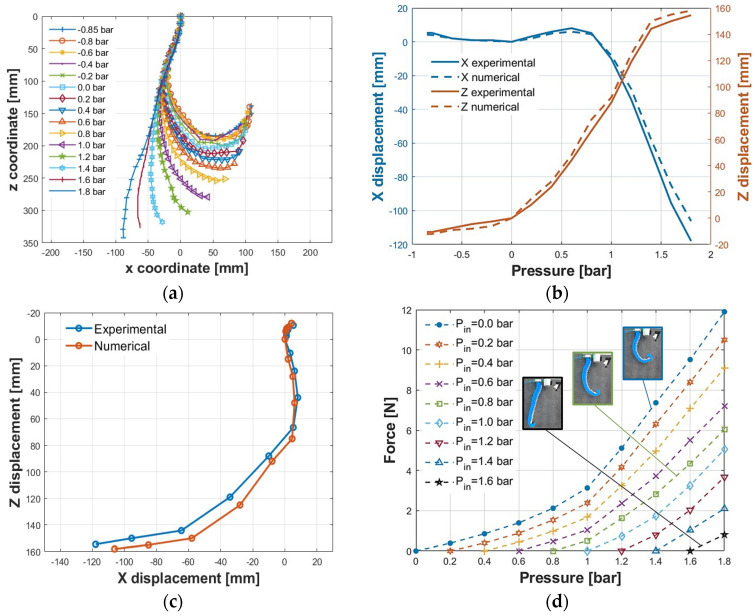
Kinematic and dynamic behavior of the actuator: (**a**) displacement of the sector markers at different feeding pressure values; (**b**) positions along the X and Z axes of point P of the actuator; (**c**) moving experimental and numerical centrode of point P; (**d**) isometric test results.

In the presence of only gravitational load, the deformation of the actuator was evaluated as a function of the pressure in the range of −0.85–1.8 bar for a total amount of fifteen pressure values. For each test, the pressure was adjusted using the precision pressure regulator, and a picture was acquired by a 12-Mpixel camera placed one meter from the actuator to limit distortion effects. An image analysis was carried out in the Solid Edge 2024 software environment to achieve the displacement of markers pointed in correspondence with the midpoint of each sector along the spiral describing the feeding channel (Figure 10a). The different deformations of the actuator are reported in Figure 11. The results of the image analysis on the kinematics of the actuator are reported in Figure 12a.

In addition, by the cobot, it was possible to evaluate the displacement of the lowest point P (Plot 6 of Figure 11) of the actuator for each pressure value. Indeed, for each of the configurations, the lowest point in contact with the load cell was brought with the cobot to acquire the X and Z coordinates. Figure 12b shows the trends of displacement along X and Z as a function of the pressure. Regarding the experimental X coordinates, for the negative values of pressure, the displacement is rather small, going from +5.2 mm to 0 mm for a pressure of −0.85 to 0 bar; meanwhile, for positive values, the displacement moves toward a positive value up to 0.8 bar (the maximum positive displacement is equal to 8 mm at 0.6 bar). At about 0.9 bar, the X coordinate of P is equal to the one at rest; hence, with an almost linear behavior, the displacement moves toward negative values with the increase in the pressure. The maximum displacement is equal to −117.9 mm at 1.8 bar. In terms of the experimental Z coordinates, for the negative values of pressure, the displacement is rather small, going from −10.5 mm to 0 mm for a pressure of −0.85 to 0 bar; meanwhile, for positive values, the displacement is interesting, reaching 144.1 mm for 1.4 bar and 154.5 mm for a pressure of 1.8 bar. In the same figure, comparisons between experimental results and numerical data are reported. The maximum absolute error is 11.81 and 8.42 mm for X and Z displacements, corresponding to 9.08% and 4.67% concerning the overall displacement range, respectively. Simulations show a lower deformation along the X direction and a higher one along Z. Such differences could depend on the viscoelastic behavior of the silicone rubber not considered in the numerical model, the dimensions of the molds, the core affected by 3D-printing tolerances, the realization process of the SPA, and/or errors introduced by image analysis. However, such a result was considered acceptable for validating the numerical model.

By combining the X and Z displacement to obtain the moving experimental and numerical centrode, the curves shown in Figure 12c were obtained. It can be noticed that over 0.9 bar, the actuator moves backward, increasing the height from the ground. Over this pressure value, both X and Z coordinates follow an almost linear behavior, which is interesting as a range for the purpose of the actuator, developing a controller and avoiding working at too high pressures that would result in excessive stresses, limiting the actuator’s useful life. It is interesting to observe how the actuator seems to follow the behaviour of a seahorse tail.

### 4.2. Isometric Tests

The force exerted by the actuator was achieved as a function of the pressure value, for a given initial deformation. Except for the negative pressure range, where the actuator cannot provide for a pushing force, before each test, the actuator was set to an initial deformation corresponding to one of the pressure values between 0 and 1.6 bar with steps of 0.2 bar, as shown in Figure 11. Tests were carried out approaching point P of the actuator to the contact plate of the load cell, adjusting the position by the cobot (Figure 10b). Each test was carried out three times showing maximum deviations of ±0.2 N, thus showing good reproducibility.

The force curves developed for different initial pressures (equivalently initial deformations) are shown in Figure 12c. The maximum achieved force is 11.9 N for a pressure of 1.8 bar, starting from an initial pressure of 0 bar, while at the same pressure value, it drops to 0.8 N for an initial pressure of 1.6 bar. Therefore, when initializing the actuator with a higher initial pressure, the forces that develop decrease because more strain energy is required.

### 4.3. Activation/Deactivation Times

**Figure 13:**
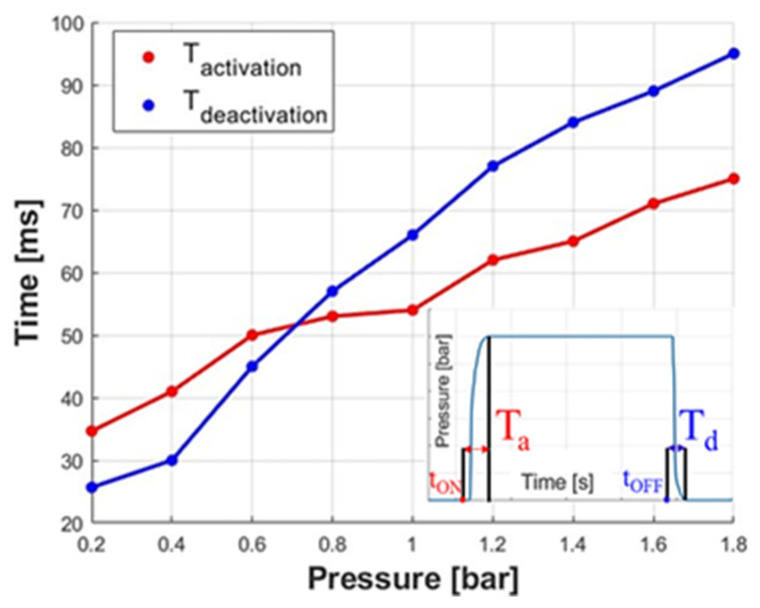
Time tests: activation and deactivation times as a function of the feeding pressure.

Both times were evaluated by setting the operating pressure with the precision pressure regulator and managing, by the 3/2 valve, the instants at which to give t_ON_ for starting the extension of the actuator or take off t_OFF_ for starting the relaxation. The activation time T_a_ is defined as the interval between t_ON_ and the instant at which the reference pressure is reached, whereas the deactivation time T_d_ is the interval between t_OFF_ and the reaching of the environmental pressure, as shown in Figure 13. Both times increase with operating pressure. In the 0–1.8 bar range, the activation and deactivation times increase from 34.7 to 75.1 ms and from 25.7 to 94.8 ms, respectively.

## 5. Conclusions

In this paper, a novel SPA inspired by the tail of the seahorse *Hippocampus reidi* was presented. The actuator developed is light, economical, and simple to realize by combining a 3D-printed reinforcement in TPU, mimicking the skeletal apparatus, with R PRO20 silicone rubber coating, replicating the functions of epithelial tissue. A pneumatic channel interacting with the coating acts as the muscle for the motion of the tail. The process of making the actuator itself is innovative and allows intimate integration between the reinforcement structure and the coating, as is the case for several biological structures in nature. The pneumatic actuation ensures safe modulation of extension or flexion, avoiding the use of electric motors and thus burdening the device.

Indeed, the realized prototype was characterized by isotonic tests for a null external load, isometric tests, and activation/deactivation times in the range of 0–1.8 bar. The tests demonstrate the possibility of using the actuator as a leg to support a multi-legged robot. Indeed, the displacement of the actuator–ground contact point shows an almost linear characteristic in the 0.9–1.8 bar range. The maximum developed force of 11.9 N is seen at 1.8 bar. Pressure values over 1.2 bar are sufficient to support the actuator itself and integrate it into a more complex structure. Activation and deactivation times are less than 100 ms, so with an appropriate controller, it is possible to have a ready and responsive system.

Comparisons between experimental results and numerical data are reported. The maximum absolute error is 11.81 and 8.42 mm for X and Z displacements, corresponding to 9.08% and 4.67% concerning the overall displacement range, respectively. Such differences could depend on the viscoelastic behaviour of the silicone rubber not considered in the numerical model, the dimensions of the molds, the core affected by 3D-printing tolerances, the realization process of the SPA, and/or errors introduced by image analysis.

Further studies will be conducted to have strain state control not only for ventral deformation but also according to more complex profiles, such as overstretching and helical bending, by more pneumatic feeding channels in parallel.

Moreover, this work could contribute to the development of new technologies, such as prehensile robots or new types of terminal devices. The process presented, on the other hand, could help the integration of skeletal structures and epithelial tissues.

## Data Availability

Data are contained within the article.
